# The relationship between teachers’ cognitive flexibility and perceptions of curriculum change: the mediating role of innovative behavior

**DOI:** 10.3389/fpsyg.2026.1810957

**Published:** 2026-06-30

**Authors:** Alper Aytaç

**Affiliations:** Ministry of National Education, Gürsu Science and Art Center, Bursa, Türkiye

**Keywords:** cognitive flexibility, innovative behavior, mediating role, perceptions of curriculum change, teachers

## Abstract

**Background:**

Curriculum reforms require teachers not only to understand new curricular expectations but also to adapt them to classroom practice. In this process, teachers’ cognitive resources and professional behaviors may be associated with how they interpret and respond to curriculum change. Although cognitive flexibility, innovative behavior, and perceptions of curriculum change have been examined separately in previous research, limited attention has been paid to how these constructs are jointly related within a single explanatory model.

**Aim:**

This study aims to examine the mediating role of innovative behavior in the relationship between teachers’ cognitive flexibility and their perceptions of curriculum change.

**Method:**

A cross-sectional correlational survey design was employed in the study. The sample consisted of 324 teachers working in a city in Türkiye during the 2025–2026 academic year and selected through convenience sampling. The Cognitive Flexibility Inventory, the Perception of Curriculum Change Scale, and the Innovative Behavior Scale were used as data collection instruments. SPSS PROCESS Macro version 5.0 was used for data analysis. In this context, the Errors-in-Variables (EIV) approach, which accounts for measurement error and may provide less biased parameter estimates, was adopted. In addition, the bootstrap method with 5,000 resamples and 95% confidence intervals was used to evaluate the indirect effect.

**Results:**

Teachers’ cognitive flexibility was positively and significantly associated with both their perceptions of curriculum change and their innovative behavior. Teachers’ innovative behavior was also positively and significantly associated with their perceptions of curriculum change. The mediation analysis revealed a significant indirect relationship between teachers’ cognitive flexibility and their perceptions of curriculum change through innovative behavior. Since the indirect effect was significant while the direct relationship became non-significant after innovative behavior was included in the model, the findings indicated an indirect-only mediation pattern.

**Conclusion:**

This study suggests that innovative behavior helps explain the relationship between teachers’ cognitive flexibility and their perceptions of curriculum change. Teachers with higher levels of cognitive flexibility may be more likely to report higher levels of innovative behavior, which in turn is associated with more positive perceptions of curriculum change.

## 1 Introduction

Cognitive flexibility refers to an individual’s ability to adapt rapidly to changing environmental conditions and to generate alternative solutions to problem situations. In this process, individuals can develop different perspectives and make evaluations regarding newly encountered situations (Dennis and Vander Wal, 2010; Spiro and Jehng, 1990). When considered in the context of teaching, cognitive flexibility may have significant implications (Stein et al., 2018). Cognitive flexibility may enable teachers to produce appropriate pedagogical solutions to complex situations encountered in teaching–learning environments and to conduct the instructional process in accordance with students’ individual differences and needs (Martin and Rubin, 1995; Morales and Baguio, 2025). In this respect, cognitive flexibility may be considered a critical construct for adapting to educational innovations and responding positively to curriculum changes. Curricula are continuously updated, and teachers are expected to adapt to these changes. Within this context, teachers’ cognitive flexibility may facilitate perceiving curriculum changes as opportunities for development rather than as threats (Dumbi and Indrasari, 2024a).

Perceptions of curriculum change refer to how teachers evaluate new curricula and the extent to which they perceive these changes as necessary, feasible, and useful (Kayır and Toraman, 2021; März and Kelchtermans, 2013). While changes in curricula may be perceived by some teachers as sources of uncertainty, confusion, and additional workload (Fullan, 2007; Dikgale and Chauke, 2024), teachers who are cognitively more flexible may view such changes as more manageable. As a result, these teachers may experience the adaptation process to curriculum changes more positively and efficiently (Idawati et al., 2024). From this perspective, cognitive flexibility may be regarded as an important psychological resource that may shape perceptions of curriculum change. Particularly in light of changing instructional technologies, learner profiles, and instructional goals, the importance of teachers’ adaptive cognitive skills has become increasingly evident (Kleickmann et al., 2016; Mena-Guacas et al., 2025).

Another variable that helps to better understand the relationship between cognitive flexibility and perceptions of curriculum change is innovative behavior. Innovative behavior is defined as an individual’s generation of new ideas in the workplace, the implementation of these ideas, and engagement in initiatives aimed at improving existing processes (Scott and Bruce, 1994). Teachers who possess an innovative perspective and demonstrate such performance may perceive curriculum changes more positively and integrate innovations into classroom practices more rapidly (Alagöz and Canlı, 2024; Runhaar et al., 2010; Runhaar et al., 2016). Teachers with higher cognitive flexibility may be better positioned to generate a greater number of alternative solutions, perceive changing curricula as more applicable, and are therefore more likely to engage in innovative behaviors (Dumbi and Indrasari, 2024a). Accordingly, teachers’ innovative behaviors can be viewed as a mechanism that may strengthen the relationship between cognitive flexibility and perceptions of curriculum change.

The theoretical rationale of this study is grounded in Social Cognitive Theory and teacher agency perspectives. Social Cognitive Theory and teacher agency perspectives were selected as the guiding theoretical lenses because they jointly account for both the cognitive and behavioral dimensions of teachers’ professional responses to curriculum change, dimensions that neither framework addresses comprehensively on its own. Social Cognitive Theory emphasizes the reciprocal relationship among personal cognitive factors, behavior, and environmental conditions (Bandura, 1986, 2001). From this perspective, cognitive flexibility can be conceptualized as a personal cognitive resource that may help teachers reinterpret changing instructional demands and generate alternative responses. Innovative behavior, in turn, represents a distinct behavioral construct through which such cognitive resources may be translated into professional practice by generating, promoting, and implementing new ideas (Janssen, 2000; Scott and Bruce, 1994). In this model, innovative behavior is not treated as a mere extension or byproduct of cognitive flexibility; rather, it is conceptualized as a theoretically distinct but functionally related construct that serves as a behavioral pathway connecting cognitive resources to perceptions of curriculum change. This theoretical perspective is particularly relevant to curriculum change because educational reforms require teachers not only to understand new curriculum expectations but also to translate them into classroom practices (Remillard, 2005; Spillane et al., 2002). Teacher agency perspectives further suggest that teachers are not passive implementers of curriculum reforms; rather, they actively interpret, negotiate, and enact curriculum changes within their professional contexts (Eteläpelto et al., 2013; Priestley et al., 2015). Similarly, teacher agency models in professional development and school reform position teachers as active professional actors whose actions are shaped by dynamic relationships among individual, school-level, and broader systemic conditions (Imants and Van der Wal, 2020). Accordingly, this study assumes that innovative behavior may function as a behavioral mechanism through which teachers’ cognitive flexibility is associated with their perceptions of curriculum change. While other personal resources such as self-efficacy or emotional regulation may also mediate this relationship, innovative behavior is particularly relevant in the context of curriculum change because it directly involves the generation and implementation of new ideas in response to changing professional demands, a process that is both cognitively driven and contextually enacted.

In conclusion, the dynamic relationship among cognitive flexibility, innovative behavior, and perceptions of curriculum change provides a critical framework for understanding teachers’ ability to adapt to changing educational conditions. Therefore, examining the mediating role of innovative behavior may offer a more comprehensive understanding of the factors shaping teachers’ perceptions of curriculum change. Although previous studies have examined cognitive flexibility, innovative behavior, and perceptions of curriculum change separately, limited attention has been paid to how these constructs operate together within a single explanatory model. In this respect, the present study seeks to contribute to the literature by examining innovative behavior as a potential behavioral mechanism linking teachers’ cognitive flexibility to their perceptions of curriculum change. The findings may provide useful insights for curriculum designers, policymakers, and teacher educators seeking to support teachers’ adaptation to curriculum change.

## 2 Literature review

### 2.1 Theoretical framework: Social Cognitive Theory and teacher agency

This study draws on Social Cognitive Theory and teacher agency perspectives to explain how teachers’ cognitive characteristics may be associated with their professional responses to curriculum change. These two frameworks were selected because they jointly address both the cognitive and behavioral dimensions of teachers’ engagement with curriculum reform, dimensions that are theoretically complementary yet rarely integrated within a single explanatory model. Social Cognitive Theory conceptualizes human functioning as an interaction among personal factors, behavior, and environmental conditions (Bandura, 1986, 2001). In this framework, teachers are not simply shaped by external reform demands; rather, they interpret these demands through their cognitive resources and respond to them through professional actions. Cognitive flexibility can therefore be positioned as a personal cognitive resource that may support teachers in interpreting changing instructional expectations, considering alternative pedagogical responses, and regulating their professional practices in dynamic educational contexts (Martin and Rubin, 1995; Spiro et al., 1992). From this perspective, innovative behavior represents a theoretically distinct but functionally related behavioral construct through which such cognitive resources may become visible in practice (Janssen, 2000; Scott and Bruce, 1994). In this model, innovative behavior is not conceptualized as a mere extension of cognitive flexibility; rather, it operates as a distinct construct that serves as a behavioral pathway through which cognitive resources are translated into professional action.

Teacher agency perspectives complement this view by emphasizing teachers’ active role in curriculum implementation. Agency is not merely an individual trait; it emerges through the interaction between teachers’ capacities, professional purposes, and the contextual conditions in which they work (Biesta and Tedder, 2007; Emirbayer and Mische, 1998; Eteläpelto et al., 2013). In curriculum reform contexts, teachers make sense of policy messages, interpret new curricular expectations, and enact changes in relation to their classroom realities (Priestley et al., 2015; Spillane et al., 2002). This sense-making process is inherently cognitive in nature, suggesting that teachers’ personal cognitive resources, including their flexibility in thinking, may shape both what they perceive in curricular change and how they respond to it (März and Kelchtermans, 2013). While other mediating constructs such as self-efficacy or emotional regulation could theoretically link cognitive flexibility to curriculum change perceptions, innovative behavior is particularly relevant in this context because it specifically involves the generation, promotion, and implementation of new ideas in response to changing professional demands, making it both cognitively grounded and contextually enacted. Therefore, curriculum change can be understood as a process that depends not only on policy design but also on teachers’ cognitive and behavioral engagement with change. Within this framework, innovative behavior is conceptualized as an agentic behavioral mechanism through which cognitively flexible teachers may transform curriculum changes into more applicable and meaningful classroom practices (Bandura, 1986; Eteläpelto et al., 2013). This interpretation is also consistent with views that conceptualize teacher agency as closely intertwined with professional practice, reflection, and teachers’ capacity to make informed pedagogical decisions within their working contexts (Molla and Nolan, 2020). Accordingly, the proposed model integrates Social Cognitive Theory and teacher agency perspectives by linking teachers’ cognitive flexibility, innovative behavior, and perceptions of curriculum change within a single explanatory framework.

### 2.2 Cognitive flexibility

Cognitive flexibility is generally considered a core component of executive functioning defined as an individual’s ability to adapt to changing environmental demands, process information in multiple ways, and develop alternative strategies during problem-solving processes (Dajani and Uddin, 2015; Diamond, 2013). This process involves not only processing existing knowledge but also transforming prior knowledge and adapting it to new situations when conditions change (Scott, 1962). It can be stated that cognitive flexibility is associated with the ability to consider different perspectives, interact with new information, and implement the behavioral adjustments required by new information (Martin and Rubin, 1995). In addition, cognitive flexibility has been linked to lower resistance to change and more adaptive responses to changing conditions (Chung et al., 2012). Dennis and Vander Wal (2010) conceptualized cognitive flexibility within the context of individuals’ capacity to cope with problems and to orient toward alternative solutions. This perspective positions cognitive flexibility not only as a cognitive process but also as a critical resource in adaptation, stress management, and decision-making processes. In line with the theoretical framework of the present study, cognitive flexibility may therefore be regarded as a personal cognitive resource that may shape how individuals interpret and respond to changing environmental and professional demands.

From the perspective of the teaching profession, the importance of cognitive flexibility becomes even more evident. Diverse learning needs, student diversity, and unexpected situations in classroom teaching–learning environments require continuous cognitive and behavioral adaptation on the part of teachers. Teachers with high levels of cognitive flexibility may be better positioned to approach classroom problems from a broader perspective, respond more constructively to individual differences among students, and adapt their instructional decisions according to situational demands (Corno, 2008; Parsons et al., 2018). Such teachers may adopt multidimensional perspectives when seeking solutions to classroom challenges (Stein et al., 2018) and therefore may manage instructional processes in a more adaptive and context-sensitive manner (Cevahir Bolat and Arslan, 2024). Moreover, research indicates that cognitive flexibility is positively associated with teachers’ classroom management (Gecikli and Ak, 2024), technology integration (Ertmer and Ottenbreit-Leftwich, 2010), instructional skills (Corno, 2008), mindfulness and self-efficacy beliefs (Bekirler and Bilaloğlu, 2022), job satisfaction (Üzümcü and Müezzin, 2018), and teacher professionalism (Kaçay et al., 2021). In this context, cognitive flexibility may be regarded as a fundamental cognitive resource that may shape teachers’ competence in teaching–learning processes. This conceptualization also provides a basis for examining how teachers’ cognitive flexibility may be associated with their perceptions of curriculum change and their innovative professional behaviors.

### 2.3 Innovative behavior

Innovative behavior is generally defined as individuals’ generation of new ideas in their workplaces, the implementation of these ideas, and efforts to promote change (Janssen, 2000). One of the concrete indicators of individuals’ innovative behavior is their engagement in creative solutions and actions (West and Farr, 1990). Teachers’ innovative behavior includes actions such as implementing new instructional methods in the teaching–learning environment, effectively integrating technology into the process, diversifying instructional materials, and producing creative solutions aimed at improving students’ learning processes (Kart and Parlar, 2022; Thurlings et al., 2015). In this regard, teachers who exhibit innovative behavior may develop new methods, processes, or products that contribute to the development of their school work environments (Messmann and Mulder, 2015). Within the theoretical framework of the present study, innovative behavior may also be understood as a distinct but functionally related behavioral construct through which teachers’ cognitive and professional resources are reflected in practice. In other words, it reflects how teachers transform new ideas, alternative perspectives, and adaptive responses into concrete instructional practices.

The dissemination of innovative behavior in schools may support teachers’ professional development (Thurlings et al., 2015). Teachers’ adoption of an innovative mindset may emerge as a factor supporting students’ acquisition of 21st-century skills (Voogt and Pareja Roblin, 2012). In other words, teachers’ innovative behavior may be related to improvements in classroom dynamics and, consequently, students’ learning outcomes (Baharuddin et al., 2019). Furthermore, studies reveal that teachers’ innovative behavior is positively associated with proactive behaviors (Ertürk, 2023), self-efficacy (Dumbi and Indrasari, 2024b; İmamoğlu et al., 2021), perceived organizational support (Akkoç et al., 2023), teacher collaboration (Doğan, 2024), and cognitive flexibility (Dumbi and Indrasari, 2024a). In this context, teachers’ innovative behavior may be considered an important professional behavior associated with both organizational processes at the institutional level and teachers’ individual professional development. This perspective is consistent with teacher agency perspectives, which emphasize that teachers actively interpret, adapt, and enact educational changes within their own professional contexts. Therefore, innovative behavior is particularly relevant to curriculum change because it represents a practical pathway through which teachers may translate curriculum expectations into classroom-level action.

### 2.4 Perceptions of curriculum change

Curriculum refers to comprehensive plans designed to ensure the effective implementation of educational activities (Ornstein and Hunkins, 2018). Because curricula are shaped by changing knowledge structures, social expectations, educational policies, and instructional needs, they are periodically revised to respond to emerging educational demands (Young, 2008, 2013). Teachers play a central role in the implementation of curriculum changes. Therefore, how teachers perceive these changes is of particular importance (Fullan, 2007; Janko and Pešková, 2017). Teachers’ perceptions of curriculum change relate to how they make sense of curricular innovations and the extent to which they perceive these changes as necessary, feasible, and beneficial (Kayır and Toraman, 2021; März and Kelchtermans, 2013). In line with teacher agency and sense-making perspectives, these perceptions are not merely passive reactions to reform policies; rather, they reflect teachers’ interpretations of curricular expectations within their own professional and classroom contexts (Priestley et al., 2015; Spillane et al., 2002).

Teachers’ perceptions of curriculum change are crucial for the meaningful and context-sensitive implementation of curricular reforms (Bantwini, 2010; Park and Sung, 2013). Teachers’ positive attitudes toward curriculum change may support the effective implementation of curriculum reforms (Fullan, 2007). However, changes introduced through curriculum reforms are not always welcomed positively by teachers. Uncertainty arising from changes, increased workload, and insufficient support for implementation may lead teachers to develop negative attitudes toward change (Janko and Pešková, 2017; Kwok, 2014). Indeed, Kayır and Toraman (2021) emphasize that uncertainty, implementation-related difficulties, and increased workload are among the most prominent factors contributing to teachers’ negative perceptions of changing curricula. Thus, teachers’ perceptions of curriculum change constitute an important psychological and professional indicator for understanding how curriculum reforms are interpreted, negotiated, and enacted in classroom practice. Despite the growing body of research on these constructs individually, limited research has examined cognitive flexibility, innovative behavior, and perceptions of curriculum change together within a single integrative explanatory model. The present study addresses this gap by proposing and empirically testing innovative behavior as a behavioral mechanism linking teachers’ cognitive flexibility to their perceptions of curriculum change.

## 3 Research hypotheses

In order to provide a concrete indication of changes occurring in education, it is necessary to focus on what teachers think about these changes and how they implement them in practice. Teachers’ perceptions are of critical importance in this regard (Fullan, 2007). Accordingly, teachers’ perceptions of curriculum change play a crucial role in the implementation of curriculum reforms in practice (Park and Sung, 2013). In response to such changes, teachers may react in different ways, ranging from active engagement and adaptation to hesitation or resistance (Kayır and Toraman, 2021). Teachers are often expected to adapt to curricular changes within a relatively limited period of time (Chimbi and Jita, 2019). At this point, the concept of cognitive flexibility becomes salient. Cognitive flexibility refers to individuals’ capacity to adapt their thinking and behavior to changing conditions, to recognize alternative courses of action, and to evaluate situations from multiple perspectives (Dajani and Uddin, 2015). Research has shown that individuals with higher levels of cognitive flexibility may exhibit lower resistance to change (Chung et al., 2012; Uras et al., 2025). From a social cognitive perspective, personal cognitive resources shape how individuals interpret and respond to changing contextual demands (Bandura, 1986, 2001). In this framework, cognitive flexibility may be positioned as a cognitive resource that may enable teachers to reframe curricular demands, consider alternative pedagogical responses, and approach change-related uncertainty in a more adaptive manner. This interpretation is also consistent with recent research on curriculum literacy and cognitive flexibility. Can Yurt and Saracaloğlu (2026) found a moderate and significant positive relationship between teachers’ curriculum literacy self-efficacy perceptions and their cognitive flexibility levels. This finding is meaningful because curriculum literacy is closely related to teachers’ ability to understand, interpret, adapt, and implement curricula in classroom contexts. Therefore, cognitive flexibility may support teachers not only in responding to change in general but also in making sense of curriculum-related expectations and adapting them to classroom practice. Indeed, the study by Idawati et al. (2024) provides empirical support for this argument, demonstrating that teachers with higher cognitive flexibility reported greater readiness to implement new curricula. Based on this rationale, the following hypothesis is proposed:

*H1*: Teachers’ cognitive flexibility is positively associated with their perceptions of curriculum change.

Innovative behavior generally refers to individuals’ generation of creative ideas, their ability to recognize problem situations, and their capacity to advocate for and support the ideas they produce (Janssen, 2000; Scott and Bruce, 1994). In the educational context, teachers’ innovative behavior encompasses their ability to integrate creative ideas into the classroom environment. Examples of such behavior include experimenting with new instructional strategies, enriching lesson design, and integrating technology into classroom practices (Runhaar et al., 2016). In this respect, it can be argued that being open to change may be an important condition for teachers to exhibit innovative behavior, and this openness may be closely related to cognitive flexibility. Cognitive flexibility is defined as the capacity to rapidly reorganize mental processes in response to changing conditions and to generate appropriate responses within this restructuring process (Braem and Egner, 2018). This capacity may be considered a cognitive foundation for the idea generation and implementation stages of innovative behavior (Hohl and Dolcos, 2024). Social Cognitive Theory further supports this link by suggesting that personal cognitive factors shape behavioral patterns through processes of self-reflection, forethought, and self-regulation (Bandura, 1986, 2001). In other words, teachers who are cognitively flexible may be better positioned to evaluate existing practices critically, anticipate alternative instructional possibilities, and regulate their professional actions in ways that are reflected in innovative behavior. The findings reported by Dumbi and Indrasari (2024a) also provide evidence for this relationship, indicating that teachers’ cognitive flexibility was positively associated with their innovative behavior. Accordingly, the following hypothesis is proposed:

*H2*: Teachers’ cognitive flexibility is positively associated with their innovative behavior.

Innovative teachers may follow current developments in the field of education in order to enhance their professional competencies and to implement these developments in the classroom (Lambriex-Schmitz et al., 2020). In other words, as teachers’ levels of innovative behavior increase, they may be more likely to respond constructively to changing educational paradigms. This interpretation is consistent with organizational behavior research showing that innovative behavior is associated with change-related orientations, such as commitment to change and organizational support for creativity (Jun and Lee, 2023). From a teacher agency perspective, innovative behavior reflects teachers’ active role in interpreting and enacting curriculum change rather than merely complying with externally imposed reform directives (Eteläpelto et al., 2013; Priestley et al., 2015). Teachers who engage in innovative behavior may be more likely to perceive curriculum changes as professionally meaningful and practically applicable, because their orientation toward generating and implementing new ideas aligns with the demands of reform-oriented practice. Similarly, Runhaar et al. (2016) suggest that developments in science and technology may encourage teachers to be open to innovation. In this context, it can be argued that innovative teachers may be more likely to approach curriculum changes positively. Changes in curricula are accompanied by shifts in instructional, assessment, and evaluation processes, which in turn reshape classroom dynamics. Adapting to such processes may require teachers to be open to innovation. Dumbi and Indrasari (2024a) further assert that teachers’ innovative behavior plays a key role in ensuring that curriculum changes within comprehensive educational reforms are effectively reflected in teaching-learning environments. Thus, innovative behavior may serve as a practical and agentic response through which teachers make curriculum changes more applicable in classroom practice. Based on this reasoning, the following hypothesis is proposed:

*H3*: Teachers’ innovative behavior is positively associated with their perceptions of curriculum change.

Curriculum changes yield outcomes not only through the revision of policy documents but also through how teachers make sense of change and translate it into practice within the teaching–learning process (März and Kelchtermans, 2013; Spillane et al., 2002). In this respect, the success of educational change may be considered to be closely related to teachers’ cognitive and professional capacity to make sense of curriculum changes, adapt to them, and enact them in practice (Bümen and Holmqvist, 2022; Spillane, 1999; Yakavets et al., 2023). Social Cognitive Theory and teacher agency perspectives suggest that cognitive resources may be reflected in professional actions, and that such actions may be associated with how teachers interpret and enact curriculum change (Bandura, 1986, 2001; Priestley et al., 2015). According to Janssen (2000), innovative behavior involves generating, promoting, and implementing new ideas in practice. From this perspective, an innovative orientation may support teachers’ adaptation to change by helping them translate new ideas into concrete professional practices. In this integrated framework, cognitive flexibility represents a personal cognitive resource, whereas innovative behavior represents a theoretically distinct but functionally related behavioral construct through which this resource is enacted in professional practice. Innovative behavior is particularly relevant as a mediating mechanism in this context because it links teachers’ cognitive capacity to professional action in response to changing curricular demands. While other constructs such as self-efficacy or emotional regulation could theoretically serve as mediators, innovative behavior is uniquely positioned at the intersection of cognitive capacity and professional action in curriculum reform contexts. Accordingly, it can be argued that innovative behavior may be associated with how teachers with higher levels of cognitive flexibility perceive curriculum changes. Based on this rationale, the following hypothesis is proposed:

*H4*: Innovative behavior mediates the relationship between teachers’ cognitive flexibility and their perceptions of curriculum change.

## 4 Method

### 4.1 Design

This study employed a cross-sectional correlational survey design. The correlational design was selected to examine the mediating role of innovative behavior in the relationship between teachers’ cognitive flexibility and their perceptions of curriculum change. Since the data were collected from participants at a single point in time, the study does not allow causal inferences. Therefore, the proposed mediation model was tested to examine theoretically grounded associations and indirect relationships among the variables rather than to establish causal effects.

### 4.2 Sample

The sample of the study consisted of 324 teachers working in a city in Türkiye during the 2025–2026 academic year. Convenience sampling was used to select the participants. Convenience sampling is a non-probability sampling technique in which participants are selected based on their accessibility and willingness to participate, and it is commonly used when researchers aim to reach available participants within practical field conditions (Etikan et al., 2016). This sampling method was preferred because it provided access to teachers who were available and willing to participate in the study during the data collection period. The sample included teachers from different school types and with varying levels of professional seniority, which allowed the study to include participants with diverse professional backgrounds. However, since the sample was selected through a non-probability sampling method and was limited to a single city, the findings should be interpreted with caution in terms of generalizability. Descriptive information regarding the sample is presented in Table 1.

**Table 1:** Demographic characteristics of the research sample.

Variable	Category	N	%
Gender	Female	191	59
	Male	133	41
Professional seniority	1–10 years	89	27.5
	11–20 years	126	38.9
	21 year or more	109	33.6
School type	Primary school	112	34.6
	Middle school	127	39.2
	High school	85	26.2

Table 1 presents descriptive information regarding the study sample. Accordingly, 191 teachers (59%) were female and 133 (41%) were male. Regarding professional seniority, 89 teachers (27.5%) had 1–10 years of experience, 126 (38.9%) had 11–20 years, and 109 (33.6%) had 21 years or more. In terms of school level, 112 teachers (34.6%) were working in primary schools, 127 (39.2%) in middle schools, and 85 (26.2%) in high schools.

### 4.3 Data collection instruments

#### 4.3.1 Cognitive Flexibility Inventory

The scale was developed by Dennis and Vander Wal (2010) and adapted into Turkish by Gülüm and Dağ (2012). In the first stage of the adaptation process, the scale was translated into Turkish and finalized based on expert opinions. In the second stage, the scale was administered to 266 university students. Exploratory factor analysis revealed a two-factor structure explaining 49.8% of the total variance. The Alternatives subdimension consists of 13 positively worded items, whereas the Control subdimension consists of 7 negatively worded items; therefore, the items in the Control subdimension are reverse-coded during data analysis. Reliability analyses based on Cronbach’s alpha coefficients yielded values of 0.89 for the Alternatives subdimension, 0.85 for the Control subdimension, and 0.90 for the total scale. In the present study, Cronbach’s alpha coefficients were 0.90 for the Alternatives subdimension, 0.80 for the Control subdimension, and 0.86 for the total scale. Although the Turkish adaptation of the Cognitive Flexibility Inventory was conducted with university students, the Turkish form of the scale has subsequently been used in several educational studies with teacher samples in Türkiye, providing additional contextual support for its applicability in teacher-related research (Can Yurt and Saracaloğlu, 2026; Erarslan, 2023; Ertek Eroğlu and Eker, 2025; Kartal et al., 2024). In addition, Can Yurt and Saracaloğlu (2026) reported confirmatory factor analysis results indicating that the scale showed acceptable model fit in a teacher sample (e.g., CMIN/df = 2.8, RMSEA = 0.07, CFI = 0.97, NFI = 0.97).

#### 4.3.2 Innovative behavior scale

The scale was developed by Scott and Bruce (1994) and adapted into Turkish by Çalışkan et al. (2019). The adaptation process was carried out in five stages, and the final version of the scale was obtained accordingly. The adaptation study was conducted with two different adult employee samples from various sectors. The first sample consisted of 391 participants, and the second sample consisted of 619 participants. Exploratory factor analysis conducted with the first sample indicated a single-factor structure explaining 69.28% of the total variance. Confirmatory factor analysis conducted with the second sample confirmed the single-factor structure. Reliability analyses showed Cronbach’s alpha coefficients of 0.934 for the first sample and 0.911 for the second sample. In the present study, the Cronbach’s alpha coefficient was calculated as 0.857. Although the Turkish adaptation of the Innovative Behavior Scale was conducted with adult employee samples from different sectors, prior psychometric evidence with teacher samples is limited. Therefore, confirmatory factor analysis was conducted using the data obtained from the current teacher sample to provide additional construct validity evidence. Since the scale has a single-factor structure consisting of six items, two theoretically justifiable error covariances between items within the same factor were added based on the modification indices. The CFA results indicated that the single-factor structure of the scale showed acceptable to excellent model fit (CMIN/df = 2.41, CFI = 0.98, NFI = 0.98, IFI = 0.98, RMR = 0.017, and RMSEA = 0.066). These values were evaluated based on commonly used model fit criteria in confirmatory factor analysis (Hu and Bentler, 1999; Kline, 2016). In addition, the composite reliability (CR) value was 0.86, and the average variance extracted (AVE) value was 0.50, supporting the reliability and convergent validity of the scale in the current teacher sample (Fornell and Larcker, 1981; Hair et al., 2019).

#### 4.3.3 Perceptions of curriculum change scale

The scale was developed by Kayır and Toraman (2021). Initially, a literature review was conducted, followed by the collection of expert opinions. As a result of these stages, a draft scale consisting of 23 items was developed. The scale development process was conducted with two samples. Exploratory factor analysis was performed with a sample of 122 participants, resulting in a two-factor structure. The Resistance to Curriculum Implementation subdimension consists of 5 items, all of which are negatively worded and therefore reverse-coded during analysis. The Impact of Curriculum Changes on the Teaching-Learning Environment subdimension consists of 6 items. The two-factor structure explained 63.43% of the total variance. Confirmatory factor analysis further confirmed the two-factor structure. Reliability analyses indicated Cronbach’s alpha coefficients of 0.917 for the Resistance to Curriculum Implementation subdimension, 0.767 for the Impact of Curriculum Changes on the Teaching-Learning Environment subdimension, and 0.890 for the total scale. In the present study, Cronbach’s alpha coefficients were 0.722 for the Resistance to Curriculum Implementation subdimension, 0.898 for the Impact of Curriculum Changes on the Teaching-Learning Environment subdimension, and 0.812 for the total scale.

### 4.4 Data collection and analysis

In the first stage of the study, ethical approval was obtained. Subsequently, the scales were administered. Participation in the study was voluntary, and the purpose of the research was explained to the participants. The collected data were then transferred to SPSS for analysis. The normality of the data was examined by checking skewness and kurtosis values. The skewness and kurtosis values were found to be within the range of −1 to +1, suggesting that the data did not substantially deviate from normality.

Before testing the research hypotheses, the potential risk of common method bias was examined because all variables were measured using self-report scales collected from the same participants at a single point in time. For this purpose, Harman’s single-factor test, which is widely used as an initial diagnostic procedure for common method variance, was conducted (Podsakoff et al., 2003). All items from the three measurement instruments were entered into an unrotated exploratory factor analysis. The results revealed seven factors with eigenvalues greater than 1, and the first factor accounted for only 26% of the total variance. Since the first factor did not account for the majority of the variance, common method bias was not considered to be a serious threat in the present study.

To test the research hypotheses, SPSS PROCESS Macro version 5.0 was used. In this context, the Errors-in-Variables (EIV) approach recommended by Hayes et al. (2025) was adopted. This regression approach is notable for accounting for measurement error and thus may provide less biased parameter estimates in models involving measurement error. Within this approach, HC3-type standard error correction is used by default. To apply the EIV approach, it is recommended that the reliability coefficients of the independent and mediator variables exceed 0.70 (Bozkurt, 2025). In the mediation analysis, gender, professional seniority, and school type were included as control variables to examine whether the proposed indirect relationship remained robust after accounting for demographic and institutional characteristics. Since all control variables were categorical, they were dummy-coded before being entered into the model.

According to Hayes’s (2026) updated PROCESS documentation for SPSS, when the Errors-in-Variables (EIV) approach is applied in Model 4, several output options, including standardized regression coefficients and effect size indices, cannot be produced. Therefore, in the present study, the main mediation analyses and hypothesis testing were conducted using the EIV approach, which accounts for measurement error. However, because standardized coefficients and effect size indicators could not be obtained under the EIV specification, the same Model 4 was also estimated using the conventional OLS approach as a supplementary analysis. The OLS results were used only to report standardized regression coefficients and effect size indicators; the primary interpretation of the mediation model was based on the EIV results. In addition, the completely standardized indirect effect was reported as a supplementary standardized effect size indicator for the indirect effect, following Preacher and Kelley’s (2011) recommendations.

**Figure 1:**
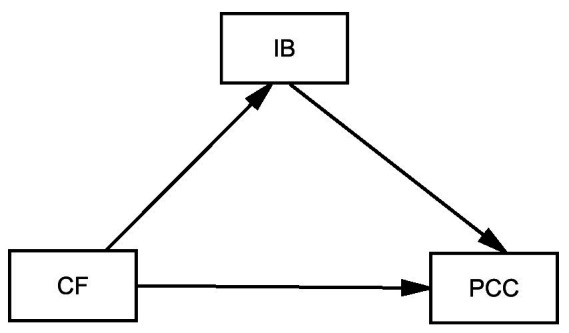
Mediation model of the relationships between variables. CF, Cognitive Flexibility; IB, Innovative Behavior; PCC, Perceptions of Curriculum Change.

The bootstrap method with 5,000 resamples and 95% confidence intervals was used to evaluate the statistical significance of the indirect effect. In bootstrap analyses, a confidence interval that does not include zero indicates a statistically significant indirect effect (Hayes, 2022). Following contemporary recommendations in mediation analysis, the interpretation focused primarily on the statistical significance and confidence interval of the indirect effect rather than categorizing mediation as “full” or “partial” in the traditional sense (Bozkurt, 2025). In addition, the pattern of the mediation results was interpreted with reference to Zhao et al.’s (2010) contemporary mediation typology. Accordingly, when the total effect and indirect effect are statistically significant but the direct effect becomes non-significant after including the mediator, the result can be described as an indirect-only mediation pattern. Therefore, the total effect, direct effect, and indirect effect were reported, but the mediation was interpreted mainly in terms of the presence and theoretical meaning of the indirect effect. The mediation model of the relationships among the variables is presented in Figure 1.

## 5 Results

Four hypotheses were tested within the scope of this study. The findings are presented in line with the order of the hypotheses. First, the findings related to the first three hypotheses are reported. Then, the findings related to the fourth hypothesis, in which the mediating variable was tested, are reported. Before presenting the hypothesis-related findings, the descriptive statistics for the main variables are provided in Table 2.

Table 2 presents the descriptive statistics for the main variables of the study. The relationships among the variables were positive, statistically significant, and moderate in magnitude. The mean scores of the variables ranged from 3.36 to 4.03. In addition, the skewness and kurtosis values ranged from −0.72 to 0.69.

**Table 2:** Descriptive statistics for the main variables.

Variables	1	2	3
1. Cognitive Flexibility (CF)	–	0.52***	0.30***
2. Innovative Behavior (IB)		–	0.41***
3. Perceptions of Curriculum Change (PCC)			–
Mean	4.03	4.22	3.36
Standard Deviation	0.44	0.58	0.69
Skewness	0.03	−0.51	0.41
Kurtosis	−0.72	−0.24	−0.19

***p < 0.001.

Table 3 presents the total effect estimates for the relationship between cognitive flexibility and perceptions of curriculum change.

**Table 3:** Regression coefficients for the relationship between cognitive flexibility and perceptions of curriculum change.

Relationship	*B*	SE(HC3)	t	*p*	*β*	η^2^
CF ➔ PCC (total effect)	0.447	0.093	4.764	< 0.001	0.287	0.079
	*F*(HC3) = 5.168	*R^2^* = 0.098				

**Figure 2:**
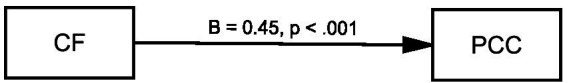
Unstandardized regression coefficient for the first hypothesis.

According to the analysis results in Table 3, a positive and statistically significant relationship was found between teachers’ cognitive flexibility and their perceptions of curriculum change (*B* = 0.447, SE(HC3) = 0.093, t = 4.764, *p* < 0.001, *β* = 0.287). The effect size (η^2^ = 0.079) was moderate. Accordingly, the first hypothesis (H₁) was supported. In addition, cognitive flexibility explained approximately 9.8% of the variance in perceptions of curriculum change (*R*^2^ = 0.098, *F*(HC3) = 5.168, *p* < 0.001). Figure 2 shows the unstandardized regression coefficient for the first hypothesis.

Table 4 presents the regression coefficients for the relationships among cognitive flexibility, perceptions of curriculum change, and innovative behavior.

**Table 4:** Regression coefficients for the relationships among cognitive flexibility, innovative behavior, and perceptions of curriculum change.

**Variables**	IB					PCC				
	*B*	SE(HC3)	*p*	*β*	η^2^	*B*	SE(HC3)	*p*	*β*	η^2^
CF	0.645	0.065	< 0.001	0.491	0.234	0.094	0.096	0.327	0.108	0.008
IB	–	–	–	–	–	0.543	0.087	< 0.001	0.363	0.089
Constant	1.841	0.286	< 0.001	–	–	0.630	0.408	0.123	–	–
	*F*(HC3) = 27.553		*p* < 0.001	*R^2^* = 0.324		*F*(HC3) = 10.194		*p* < 0.001	*R^2^* = 0.210	

**Figure 3:**
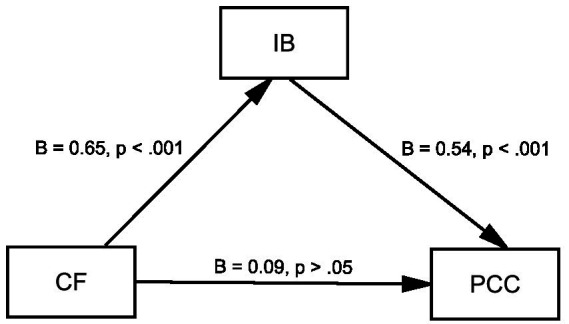
Estimated mediation model with unstandardized coefficients.

According to the analysis results in Table 4, a positive and statistically significant relationship was found between teachers’ cognitive flexibility and their innovative behavior (*B* = 0.645, SE(HC3) = 0.065, *p* < 0.001, *β* = 0.491). The effect size was large (η^2^ = 0.234). In addition, cognitive flexibility explained approximately 32% of the variance in innovative behavior (*R^2^* = 0.324, *F*(HC3) = 27.553, *p* < 001). Accordingly, the second hypothesis (H₂) was supported. A positive and statistically significant relationship was also found between teachers’ innovative behavior and their perceptions of curriculum change (*B* = 0.543, SE(HC3) = 0.087, *p* < 0.001, *β* = 0.363). The effect size was moderate (η^2^ = 0.089). Accordingly, the third hypothesis (H₃) was supported. In addition, cognitive flexibility and innovative behavior together explained approximately 21% of the variance in perceptions of curriculum change (*R^2^* = 0.210, *F*(HC3) = 10.194, *p* < 0.001). After innovative behavior was included in the model as a mediating variable, the relationship between teachers’ cognitive flexibility and their perceptions of curriculum change was found to be non-significant (*B* = 0.094, SE(HC3) = 0.096, *p* > 0.05, *β* = 0.108). The effect size was low (η^2^ = 0.008). Information regarding the model is presented in Figure 3. These results indicate that after innovative behavior was included in the model, the relationship between cognitive flexibility and perceptions of curriculum change decreased and became non-significant. The results regarding the mediating role of innovative behavior are presented in Table 5.

**Table 5:** Mediation analysis results for the role of innovative behavior in the relationship between cognitive flexibility and perceptions of curriculum change.

Effect type	Effect	BootSE	BootLLCI	BootULCI
Indirect effect	*B* = 0.353	0.063	0.238	0.487
Completely standardized indirect effect	*β* = 0.178	0.030	0.121	0.240

B represents the unstandardized indirect effect obtained from the EIV-based analysis, whereas β represents the completely standardized indirect effect obtained from the supplementary OLS analysis.

According to the results in Table 5, innovative behavior was found to play a mediating role in the relationship between teachers’ cognitive flexibility and their perceptions of curriculum change. The unstandardized indirect effect was statistically significant because the 95% percentile bootstrap confidence interval did not include zero (*B* = 0.353, BootSE = 0.063, 95% percentile bootstrap CI [0.238, 0.487]). In addition, the completely standardized indirect effect was reported as a supplementary standardized effect size indicator for the indirect effect (*β* = 0.178, BootSE = 0.030, 95% percentile bootstrap CI [0.121, 0.240]). Accordingly, the fourth hypothesis (H₄) was supported.

## 6 Discussion

In this study, the mediating role of innovative behavior in the relationship between teachers’ cognitive flexibility and their perceptions of curriculum change was examined. Within this scope, four hypotheses were tested. The discussion section is organized in line with the order of the hypotheses. In addition, the findings are interpreted through the lenses of Social Cognitive Theory and teacher agency to clarify how teachers’ cognitive resources may be translated into professional action in the context of curriculum change. From a social cognitive perspective, teachers’ perceptions and behaviors are shaped through the reciprocal interaction of personal cognitive factors, behavioral patterns, and environmental demands (Bandura, 1986, 2001). Similarly, the ecological perspective on teacher agency emphasizes that teachers do not merely implement externally prescribed curriculum changes; rather, they interpret, negotiate, and enact these changes through professional judgment within specific structural and cultural contexts (Biesta and Tedder, 2007; Priestley et al., 2015).

Within the scope of the first hypothesis, the relationship between teachers’ cognitive flexibility and their perceptions of curriculum change was examined. The analysis results indicated that teachers’ cognitive flexibility was positively and significantly associated with their perceptions of curriculum change. This finding suggests that cognitive flexibility constitutes an important cognitive resource associated with teachers’ more positive perceptions of curriculum change. When curricula change, transformations occur in many educational domains, such as classroom dynamics, teaching–learning processes, and course content (Kayır and Toraman, 2021). Teachers are expected to manage this change process effectively (Chimbi and Jita, 2019). In such contexts, cognitive flexibility may help teachers interpret new curricular expectations, consider alternative pedagogical responses, and approach uncertainty in a more adaptive manner. According to Spiro and Jehng (1990), cognitive flexibility refers to an individual’s ability to adapt rapidly to changing environmental conditions and to generate alternative solutions to problem situations. This interpretation is also consistent with Social Cognitive Theory, which views individuals as proactive and self-regulating agents rather than passive recipients of environmental demands (Bandura, 2001). Research has shown that individuals with high levels of cognitive flexibility may exhibit lower resistance to change and therefore adapt more effectively to change processes (Chung et al., 2012; Uras et al., 2025). In this context, it can be suggested that teachers with higher levels of cognitive flexibility may be more likely to respond constructively to changes in curricula (Morales and Baguio, 2025). This interpretation is also supported by recent research on curriculum literacy and cognitive flexibility. Can Yurt and Saracaloğlu (2026) found a moderate and significant positive relationship between teachers’ curriculum literacy self-efficacy perceptions and their cognitive flexibility levels. This finding is meaningful because curriculum literacy is closely related to teachers’ ability to understand, interpret, adapt, and implement curricula in classroom contexts. From this perspective, cognitive flexibility may support teachers not only in responding to change in general but also in making sense of curriculum-related expectations and adapting them to classroom practice. Indeed, the study by Idawati et al. (2024) provides evidence supporting this view, demonstrating that teachers with high cognitive flexibility reported higher levels of readiness to implement new or revised curricula. According to Dumbi and Indrasari (2024a), being cognitively flexible may help teachers to perceive curriculum changes as opportunities for development rather than as threats. From the perspective of teacher agency, this finding indicates that teachers’ cognitive flexibility may support their capacity to make professional judgments in uncertain reform contexts and to position curriculum change as a professional task that can be enacted rather than merely an external obligation (Biesta and Tedder, 2007; Priestley et al., 2015). Overall, these theoretical explanations and empirical findings support the first hypothesis of the study.

Within the scope of the second hypothesis, the relationship between teachers’ cognitive flexibility and their innovative behavior was examined. The analysis results revealed that teachers’ cognitive flexibility was positively and significantly associated with their innovative behavior. Accordingly, teachers’ cognitive flexibility may be considered an important cognitive resource related to innovative behavior. According to Janssen (2000), innovative behavior refers to the generation, promotion, and implementation of new ideas in a work context. In this context, teachers’ innovative behavior becomes tangible through their integration of creative ideas into the teaching–learning process (Kocasaraç and Karataş, 2018; Runhaar et al., 2016). From this perspective, openness to change may be regarded as an important condition for teachers’ innovative behavior, and this openness appears to be closely related to cognitive flexibility. According to Braem and Egner (2018), cognitive flexibility is defined as the capacity to rapidly restructure the mind in response to changing conditions and to generate appropriate responses during this restructuring process. This capacity may be considered to provide a cognitive foundation for the stages of idea generation and implementation inherent in innovative behavior (Hohl and Dolcos, 2024). Social Cognitive Theory further strengthens this interpretation because innovative behavior requires self-reflection, forethought, and self-regulation; that is, teachers need to evaluate existing practices, anticipate alternative possibilities, and regulate their professional actions accordingly (Bandura, 2001). In this sense, innovative behavior can be regarded as a distinct but functionally related behavioral construct through which teachers’ agentic engagement with changing instructional conditions becomes visible in practice. Research by Dumbi and Indrasari (2024a) also supports this argument, showing that teachers’ cognitive flexibility was positively related to their innovative behavior. In another study by Dumbi and Indrasari (2024b), it was found that teachers’ cognitive flexibility, together with their professional self-efficacy perceptions, was associated with their innovative behavior. Overall, the theoretical framework and related studies support the second hypothesis of this study.

Within the scope of the third hypothesis, the relationship between teachers’ innovative behavior and their perceptions of curriculum change was examined. The results of the analysis indicated that teachers’ innovative behavior was positively and significantly associated with their perceptions of curriculum change. This finding suggests that teachers who more frequently engage in innovative professional behavior may perceive curriculum change more positively. According to Lambriex-Schmitz et al. (2020), innovative teachers may follow current developments in the field of education to enhance their professional competencies and to implement these developments in classroom settings. This interpretation is consistent with organizational behavior research showing that innovative behavior is associated with change-related orientations, such as commitment to change and organizational support for creativity (Jun and Lee, 2023). In this context, considering that curriculum changes are accompanied by shifts in instructional, assessment, and evaluation processes, it can be suggested that innovative teachers may be more likely to approach these curriculum changes positively and respond to them in a more constructive and practice-oriented manner. Dumbi and Indrasari (2024a) further suggest that teachers’ innovative behavior plays a key role in ensuring that curriculum changes resulting from comprehensive educational reforms are effectively reflected in teaching–learning environments. This result can also be interpreted through teacher agency. Curriculum change becomes meaningful in practice when teachers translate policy expectations into pedagogical decisions, classroom routines, instructional materials, and assessment practices. This interpretation is consistent with Spillane’s (1999) view that external reform initiatives are reconstructed through teachers’ zones of enactment, where teachers interpret reform ideas and transform them into classroom practice within their local professional contexts. Therefore, innovative behavior may strengthen teachers’ sense that curriculum change is applicable, manageable, and professionally meaningful. However, it is important to note that teacher agency should not be reduced solely to individual willingness or personal capacity. The ecological perspective emphasizes that agency is achieved through the interaction between teachers’ personal resources and the cultural, structural, and material conditions of their professional environments (Priestley et al., 2015). Overall, the theoretical framework and related studies support the third hypothesis of this study.

Within the scope of the fourth hypothesis, the mediating role of innovative behavior in the relationship between teachers’ cognitive flexibility and their perceptions of curriculum change was examined. The analysis results revealed a significant indirect association between teachers’ cognitive flexibility and their perceptions of curriculum change through innovative behavior. This pattern is consistent with an indirect-only mediation pattern, as the indirect effect was significant while the direct effect became non-significant after innovative behavior was included in the model. In this context, teachers with higher levels of cognitive flexibility may also exhibit higher levels of innovative behavior, which in turn is associated with more positive perceptions of curriculum change. According to Kayır and Toraman (2021), teachers’ perceptions of curriculum change are largely related to their capacity to transform these changes into classroom practices. Teachers with an innovative perspective may integrate newly emerging alternative methods into their classroom practices (Lambriex-Schmitz et al., 2020). From this perspective, innovative behavior emerges as a critical mechanism that may help explain how teachers’ cognitive flexibility is reflected in their pedagogical responses to curriculum change. It is important to note that innovative behavior was not selected arbitrarily as a mediator; rather, it was theoretically grounded as a construct that is both distinct from cognitive flexibility and functionally connected to it. While other personal resources such as self-efficacy or emotional regulation could theoretically operate as mediators in this relationship, innovative behavior is particularly relevant in the context of curriculum change because it specifically entails the generation, promotion, and implementation of new ideas in response to changing professional demands, thereby making it both cognitively informed and contextually enacted (Janssen, 2000; Scott and Bruce, 1994). This mediating mechanism is particularly meaningful when considered together with Social Cognitive Theory and teacher agency. In Social Cognitive Theory, personal cognitive factors do not remain isolated from behavior; rather, they shape and are shaped by action within particular social and environmental conditions (Bandura, 1986, 2001). In the present model, cognitive flexibility represents a personal cognitive resource, innovative behavior represents a theoretically distinct but functionally related behavioral construct through which this resource is enacted, and perceptions of curriculum change reflect teachers’ evaluative orientation toward a changing professional environment. Thus, the mediation finding suggests that cognitive flexibility may become more meaningful for understanding curriculum change perceptions when it is considered together with innovative classroom-oriented action.

The finding also contributes to the teacher agency literature. Although teacher agency was not directly measured in this study, the mediation model can be interpreted as an agency-related process. Teachers with higher cognitive flexibility may be better able to reinterpret curricular demands, consider alternative pedagogical possibilities, and make professional judgments under conditions of change. However, these cognitive capacities appear to be especially meaningful when they are enacted through innovative behavior. In this respect, innovative behavior may function as a bridge between teachers’ internal cognitive resources and their active engagement with curriculum change. This interpretation aligns with the ecological view of teacher agency, which conceptualizes agency as something teachers achieve through their engagement with contextual possibilities and constraints rather than as a fixed individual trait (Biesta and Tedder, 2007; Priestley et al., 2015).

The study by Dumbi and Indrasari (2024b) showed that teachers’ cognitive flexibility was positively associated with innovative behavior and that alternative thinking capacity, in particular, was closely related to the emergence of innovative practices. Complementary evidence was reported by Idawati et al. (2024), who showed that teachers with higher cognitive flexibility reported greater readiness to implement new curricula. These findings suggest that cognitive flexibility is a fundamental cognitive resource that may help teachers to evaluate new curriculum objectives from different perspectives, move beyond traditional practices, and restructure instructional processes. Through innovative behavior, teachers may perceive and enact curriculum changes as more applicable, manageable, and meaningful, which may be associated with more positive perceptions of curriculum change. Accordingly, the findings suggest that innovative behavior helps explain an important part of the relationship between cognitive flexibility and perceptions of curriculum change. The findings of Dumbi and Indrasari (2024a) reveal a strong relationship between cognitive flexibility and innovative behavior and provide empirical support for the idea that innovative behavior may function as a behavioral mechanism through which teachers’ cognitive resources are reflected in classroom-oriented responses to curriculum change.

Finally, the inclusion of gender, professional seniority, and school type as control variables strengthens the interpretation of the main model. The persistence of the main pattern of findings among cognitive flexibility, innovative behavior, and perceptions of curriculum change after controlling for these demographic and institutional variables suggests that the proposed mechanism is not merely a reflection of basic demographic or school-type differences. Rather, teachers’ cognitive and behavioral resources appear to offer a more proximal explanation for how they evaluate curriculum change. Nevertheless, this result should not be interpreted as evidence that demographic and contextual variables are unimportant; instead, it indicates that future studies should examine these variables together with school-level factors such as leadership, professional collaboration, institutional support, and organizational climate.

## 7 Conclusion

How teachers perceive curriculum changes is important for the meaningful and context-sensitive implementation of curricula. Changes in curricula are reflected in classroom practices and, accordingly, may require changes in instructional methods and techniques. Teachers’ perceptions play a key role in ensuring that this process proceeds effectively. In this study, cognitive flexibility and innovative behavior were examined in relation to teachers’ perceptions of curriculum change, and a mediation model was proposed. Cognitive flexibility was positioned as the central cognitive construct, while innovative behavior was addressed as a behavioral mechanism linking cognitive flexibility to perceptions of curriculum change. The results revealed that teachers’ cognitive flexibility was significantly associated with both their innovative behavior and their perceptions of curriculum change.

Teachers’ innovative behavior was also significantly associated with their perceptions of curriculum change. Finally, the findings revealed a significant indirect association between teachers’ cognitive flexibility and their perceptions of curriculum change through innovative behavior. This suggests that innovative behavior helps explain how teachers’ cognitive flexibility is reflected in their perceptions of curriculum change. In this context, teachers with higher levels of cognitive flexibility may also report higher levels of innovative behavior, which in turn was associated with more positive perspectives on curriculum change. Innovative behavior was not conceptualized merely as a byproduct of cognitive flexibility; rather, it was positioned as a theoretically distinct but functionally related construct that serves as a behavioral pathway through which teachers’ cognitive resources are translated into professional responses to curriculum change. This distinction is important because it suggests that cognitive flexibility may be more meaningful for understanding teachers’ curriculum change perceptions when it is considered together with their active engagement in generating and implementing new ideas in professional practice.

The findings are interpreted within the integrated framework of Social Cognitive Theory and teacher agency perspectives. From a social cognitive standpoint, the model illustrates how personal cognitive resources, behavioral patterns, and environmental demands interact reciprocally in shaping teachers’ professional orientations toward curriculum change (Bandura, 1986, 2001). From a teacher agency perspective, the findings suggest that teachers are not passive recipients of curriculum reform; rather, their cognitive flexibility and innovative behavior collectively position them as active agents who interpret, negotiate, and enact curriculum changes within their own professional contexts (Eteläpelto et al., 2013; Priestley et al., 2015). The integration of these two frameworks contributes to the literature by offering a theoretically grounded explanation of how individual cognitive resources may be translated into agentic professional in the context of curriculum change.

The findings of the study provide important implications for teacher educators, curriculum designers, and professional development planners. Awareness-raising seminars may be organized to emphasize the importance of teachers’ adaptation to changing educational conditions. In particular, in-service training programs aimed at enhancing teachers’ cognitive flexibility may be designed. These programs may be developed as comprehensive training initiatives that include both theoretical and practical components. To increase teachers’ levels of cognitive flexibility, in-service training plans enriched with methods and techniques such as scenario-based problem solving, generating alternative solutions, case-based activities, and simulations of unexpected situations in classroom and teaching–learning environments may be prepared.

School leaders also play a critical role in supporting teachers’ adaptation to curriculum change. Principals and instructional leaders may create enabling conditions for teachers’ cognitive flexibility and innovative behavior by fostering a school culture that values professional experimentation, tolerates uncertainty, and provides structured support during reform implementation (Pak et al., 2020; Priestley et al., 2015). In particular, school leaders may facilitate collaborative professional learning environments in which teachers can share innovative practices, reflect on curriculum expectations together, and develop shared interpretations of curricular change.

At the policy level, the findings suggest that curriculum reform initiatives may be implemented more effectively when policymakers take into account teachers’ cognitive and behavioral resources rather than focusing solely on the technical dimensions of reform. This view is consistent with recent OECD work emphasizing that curriculum flexibility and autonomy are important for adapting curricula to diverse learner needs and local educational contexts (OECD, 2024). Policy designs that build in time, support structures, and professional autonomy for teachers may be more conducive to fostering the kind of cognitive flexibility and innovative engagement that the present findings suggest are associated with positive curriculum change perceptions (Fullan, 2007; Spillane et al., 2002). In this respect, teachers should be positioned not merely as recipients of curriculum policy but as active professional agents whose interpretive and innovative capacities are central to the success of reform efforts (Priestley et al., 2015).

## 8 Limitations and future research

This study has several limitations that should be considered when interpreting the findings. First, the sample consisted of teachers working in a single city in Türkiye and was selected through convenience sampling. Therefore, the findings should be interpreted cautiously in terms of generalizability. Future studies may include teachers from different regions, school contexts, and educational levels by using probability-based or more diverse sampling strategies. Furthermore, the findings should be interpreted within the specific educational and policy context of Türkiye, where curriculum reforms are predominantly designed and implemented centrally by the Ministry of National Education. Cultural, institutional, and policy-related factors specific to this context may influence the relationships among the studied variables, and caution is warranted when transferring the findings to educational systems with different curricular governance structures.

Second, the study employed a cross-sectional correlational design. Although the proposed mediation model was theoretically grounded, the design does not allow causal inferences or conclusions about temporal ordering among the variables. Future research may use longitudinal or experimental designs to examine how teachers’ cognitive flexibility, innovative behavior, and perceptions of curriculum change develop over time.

Third, all variables were measured using self-report scales collected from the same participants at a single point in time. Although Harman’s single-factor test suggested that common method bias was not a serious threat in the present study, the possibility of common method bias cannot be completely ruled out. Future studies may use multi-source data, such as administrator evaluations, peer assessments, classroom observations, or qualitative interviews, to provide richer evidence and reduce reliance on single-source self-report data.

Finally, although the measurement instruments showed acceptable internal consistency in the present sample and their original development and Turkish adaptation studies provided prior validity evidence, further validation studies with larger and more diverse teacher samples may strengthen the measurement basis of future research. In particular, additional psychometric studies may provide stronger evidence regarding the construct validity of these instruments across different teacher groups and educational contexts. Future studies may also examine additional contextual variables such as school leadership, institutional support, professional collaboration, teacher autonomy, and organizational climate to better understand the conditions under which teachers adapt to curriculum change.

## Data Availability

The raw data supporting the conclusions of this article will be made available by the author, without undue reservation.
